# C-857-08. Time for an Ethics Consult? When Burn Provider Recommendations Conflict with Parent’s Preferences

**DOI:** 10.1093/jbcr/irag033.108

**Published:** 2026-04-06

**Authors:** Marcie A Lambrix, Stacy Cantale Thomas

**Affiliations:** The MetroHealth System/Case Western Reserve University, Cleveland, OH; The MetroHealth System, Cleveland, OH

## Abstract

This interactive presentation highlights the important role a hospital’s clinical ethics service can play when trying to bridge the nuanced tensions and emotional distress that can occur when a patient’s parents (or legal guardians) medical decision making does not coincide with the team's medical recommendations. This scenario is based off the case of a Black female pediatric burn patient, 24 months old, who presented to our ABA verified burn unit with scald burns sustained after grabbing hot coffee from the counter. The accident resulted in partial thickness burns to the child’s scalp, left face, left anterior chest/shoulder/back area, totaling around 10% TBSA.

Shortly after the parents and their daughter were admitted tension regarding the team’s medical recommendations and the parent’s declination of said recommendations began to build. In this instance, the parents were declining what the team believed to be necessary IV pain management. Instead, the parents explained they wanted to manage the pain with oral medications, as they did not want to further harm their child with needles given her burns and complex medical history which included a supravalvular pulmonary valve patch plasty early in life. The team was empathetic to the parent’s reasoning, however, strongly felt the appropriate pain management were IV pain medications.

In their discomfort, the team began to question their ethical obligation as providers to intervene. The Nurse Practitioner engaged the Clinical Ethics consult service in the hopes of de-escalating tension among the team and parents, and to ideally address two specific concerns: 1) given clinicians are ethically obligated to advocate for the child’s best interests - including, in this case, appropriate relief from pain and suffering, what ethical obligation does the team have to respect the patient’s declination of IV pain medications given that in their professional opinions IV pain medication is the standard of care option, and 2). Given that parental authority is not an absolute or a given, and, in this case, it is in their professional opinions the parents are not acting in the patient’s best medical interest, is there a legal obligation to protect the child’s welfare?

